# Dr. Louis Waldstein and the Pilocarpine Treatment of Tuberculosis and Cancer

**Published:** 1895-05

**Authors:** 


Dr. Louis Waldstein, of New York, now of Berlin, Germany, will present for consideration at the coming Medical Congress of Munich, a suggested plan of treatment for tuberculosis and cancer. Specially is it directed toward the cure of lupus. Dr. Waldstein for many years has carried on experimentally the action of pilocarpine on the salivary and sweat glands, carrying out the actions noted on the various glands of the lymphatic system, and reasoning from their eliminatory power that they were stimulated powerfully by pilocarpine.

The key of his discovery is this: "By successive injections of minute doses of pilocarpine in the veins he arrives at a gradual stimulation of the lymphatic system. That system increases the white corpuscles in the blood, which corpuscles—as is well established through Metchnikoff, of the Pasteur Institute, of Paris; Hankin, of Cambridge, and Buchner, of Munich—in some way not generally agreed upon, do certainly overcome and cause to be harmless those poisonous particles in the blood which produce disease. Metchnikoff thinks that the microbes which destroy the red corpuscles of the blood are swallowed and englobed alive by the white corpuscles. Hankin and Buchner think that the white corpuscles merely absorb the dead microbes, and therefore call the white corpuscles alexine or protective particles. Dr. Waldstein goes to the fountain whence these white corpuscles spring, and tries to enliven its action and productiveness, when, through disease, these health-giving particles have become too few to keep the blood in proper order.

"Dr. Waldstein has not had time to watch the effect of his discovery in relation to tuberculosis of the lungs, but the reasoning that led him to what he has already achieved seems equally good for the cure of this terrible scourge of mankind. He strongly advises physicians to try pilocarpine in the early stages of consumption, and, indeed, in all diseases where the lymphatic system is involved, because of its stimulating action upon the organs in that system and the consequent production of white corpuscles. He has satisfied himself that pilocarpine, when injected in the veins, forms a trustworthy test for the presence of tubercular disease in man and animals, giving the physician the strongest possible certainty in the diagnosis of obscure cases.

"A striking instance of the truth of his reasoning is the case of a man, twenty-four years old, a Berliner, who has had a lupus on the back of his right hand for twenty-two years and was thought incurable. Relief was immediate after the first injection, and the hand is almost healed. This cure has created a sensation among medical men, and some hope that the road to the cure of cancer has been entered."

Dr. Louis Waldstein is a well-known physician of New York, born in that city some forty-two years ago. His father was for many years a leading optician, and his younger brother is Dr. Charles Waldstein, the archæologist, recently appointed Slade Professor at the University of Cambridge, England.

